# Digital divide among people with disabilities: Analysis of data from a nationwide study for determinants of Internet use and activities performed online

**DOI:** 10.1371/journal.pone.0179825

**Published:** 2017-06-29

**Authors:** Mariusz Duplaga

**Affiliations:** Department of Health Promotion, Institute of Public Health, Faculty of Health Sciences, Jagiellonian University Medical College, Krakow, Poland; University of Illinois at Urbana-Champaign, UNITED STATES

## Abstract

**Introduction:**

The Internet is both an opportunity as well as a challenge for people with disabilities. However, this segment of the population is usually indicated among social groups experiencing digital divide. The study is focused on the analysis of factors determining Internet usage and undertaking specific activities online among people with disabilities based on a nationwide study performed in 2013 in Poland.

**Methods:**

Secondary analysis was performed on the data of persons who declared disability status in 2013 “Social Diagnosis” study. Multivariate logistic regression models were developed for the use of the Internet and performing three types of activities online.

**Results:**

Among 3,556 respondents with disability 51.02% were females, 25.19% 65 years of age and over and 33.05% were Internet users. The predictors of Internet usage included the degree of disability, place of residence, level of education, marital status, occupational status, net income, use of health care service and the use of mobile phone. The odds ratio that a person with disability belonging to the oldest category will use the Internet was only 0.04 (95% CI 0.02–0.09), when compared to the youngest category. The odds that a person with disability from the highest category of education will use the Internet were 18 times higher than in the case of persons with only basic education (OR 18.17, 95% CI 11.70–28.21). Common predictors of online activities (accessing websites of public institutions, checking and sending emails, publishing own content on the Internet) included age category and net income.

**Conclusions:**

People with disabilities in Poland are facing a significant digital divide. The factors determining the use of the Internet in this group are similar to those of the general population. On the other hand, people with disabilities who are active online, access diversified types of services including presentation of their own content online.

## Introduction

The study aims to analyze the frequency of Internet usage and activities performed online by people with disabilities in the Polish population. Furthermore, the potential predictors of Internet usage and selected online activities were assessed. Finally, the assessment conducted in this paper aims to reveal differences in the meaning of specific predictors of Internet use and selected activities performed online.

Care for people with disabilities remains one of the greatest challenges for social and health care systems in Poland. The number of people with disabilities living in society reaches 18% [1] and may grow further in line with the ageing trend seen in most European countries. Modern societies benefit from accelerated intake of information and communication technologies (ICT) in various domains of economy and everyday life and the Internet and other ICT can be seen as an opportunity for improved support and inclusion of people with disabilities.

Unfortunately, Poland lags behind European leaders in developing information society. This process accelerated considerably after Poland joined the European Union in 2004, but still, at least 30% of citizens do not use Internet [2]. It is also clear that people with disabilities use the Internet less frequently than the general population. The potential opportunity of increasing their participation in society through ICT tools lacks fulfillment. The digital divide observed in this population is at least partially attributed to the fact that this group is composed in a great part of the elderly, who themselves experience digital divide [3]. It is also obvious that other socioeconomic factors play a significant role in limiting their access to the Internet. It should be remembered that Poland underwent radical economic changes in the early 1990s with transformation of the political system and transition to a market economy. However, catching up with other European countries benefiting from prolonged periods of democracy after II World War has not been an easy path. Moreover, not all citizens have been able to benefit from the economic transformation, especially those less educated, those living in neglected areas or suffering from other types of deprivation. After 25 years of economy transformation, Poland is now considered to be a “developed economy” according to United Nations. In retrospect over Poland’s progress in comparison to other nations still undergoing the developmental stage, the author’s review of available English literature from these nations has revealed that few studies (including those performed in Poland) have placed focus on the use of ICT by persons with disabilities. One of the main purposes of this paper is to help bridge this information gap.

The results of similar studies carried out in other countries, confirm that people with disabilities frequently experience digital divide [4–6]. So far, the determinants of this phenomenon have not been comprehensively analyzed in Poland. To some extent, both central budget resources and funding obtained from the European Union coming from so called structural funds may be used for support of this population. Developing this group’s computer skills and enabling their access to the Internet is perceived as an important objective for such programmes. A comprehensive view of facilitators and barriers for Internet use by people with disabilities should help better streamline efforts improving their e-inclusion. Identifying factors predicting Internet use among people with disabilities and assessing their importance should also help to compare the situation in Poland with other countries which are or were in a similar stage of economic transformation to benchmark possible strategies targeting Internet literacy development.

The “Social diagnosis” survey is one of the most comprehensive nation-wide panel studies focused on the assessment of quality of life and social capital in Poland [7]. This survey yields a broad scope of parameters enabling deeper insight into trends occurring in this society. The dataset from this study allows for modelling Internet usage by people with disabilities with inclusion of a broad array of variables representing sociodemographic and economic factors as well as characteristics related to the health status and disability itself.

Throughout the ages, disability has been treated as a personal misfortune and a problem causing an additional burden upon society. During the 1970s a social model of disability was proposed relying on the concept of social barriers [8–10]. According to the International Classification of Functioning, Disability and Health (ICF), disability is “an umbrella term, covering impairments, activity limitations and participation restrictions” [11]. Estimates from 2004 indicated that as much as 15.3% of the world population (978 million people) experienced some form of disability [12]. The World Health Organization (WHO) estimates also suggested that the global prevalence of disabilities in the population of 15 years or over in 2010 reached 19.4% [13].

Many available assistive technologies for people with disabilities are based on the use of ICT assuring improved communication, learning opportunities or professional activities, e.g. augmentative-alternative communication devices [14] or computer-based speech recognition and synthesis [15]. ICT are one of the key areas of development of assistive technologies.

The use of ICT may be seen both as an opportunity as well as a source of potential barriers for people with disabilities. One should remember that disability may preclude the use of information technology (IT), e.g. accessing the Internet [4]. The awareness that people with disabilities are experiencing an augmented digital divide resulted in initiatives aimed at increasing the accessibility of resources available on the Internet, e.g. the Web Accessibility Initiative Guidelines (WAIG). Technical progress has led to solutions providing support or even substituting for deficient functions in individuals with disabilities [16].

The Internet is perhaps the prime example of how such people may benefit from ICT. Some authors postulate that the Internet enables avoidance of isolation and the stigma associated with having a disability [17]. The Internet may also serve as a tool enabling or supporting professional activities. Internet access may also increase the sense of independence and self-determination in people with disabilities [18]. From a practical point of view, everyday life of individuals with disabilities may be substantially improved through access to such online services as e-banking, Internet shopping or simply communicating via e-mail or videoconferencing with families and friends [5,19–20].

For those who experience from limited mobility, the use of the Internet is sometimes the only way to perform activities which otherwise would be unavailable to them. People with communication problems due to hearing and visual impairments can benefit from the available tools which aid their sensory deficiencies. According to some reports, people with specific types of disability, e.g. blindness, may reveal a high level of computer and Internet expertise [21]. In general, ICT brings promise of empowerment for such people to reach the same degree of functionality as people without disabilities [22].

Although the Internet is a potential source of opportunities for people with disabilities, its use among this group is usually much lower than in the general population. In a report of an extensive survey published by the US Department of Commerce in 2000, it was indicated that digital divide showed a decreasing tendency in relation to the rich and poor but persisted in relation to people with a disability [5]. A survey performed in the United Kingdom demonstrated that Internet usage is disproportionately low among people with disabilities when compared with the general population [6]. According to Fox, the rate of Internet use among Americans with disabilities was 54%, and among adults with no disability as much as 81% [4]. Some authors introduced the term ‘disability divide” to name the digital gap observed in the population of people with disabilities [19].

Disabilities may increase the digital divide in affected populations due to insufficient financial resources or skills and tools which would enable them to fully benefit from Internet access. This low accessibility to Internet resources may itself be another factor limiting its use by people with disabilities. This group’s low Internet usage is related to the fact that they do not only experience the digital divide, but frequently from other types of deprivation; e.g. low socio-economic status, shortage of financial resources and dependence on family members or social support [23]. It is also obvious that the elderly population, in which disabilities are most common, reveals a much lower rate of Internet use in comparison to other age groups. In the European Union, only 43% of the elderly between 65–74 years of age, and only 20% of those 75 years and over had access to the Internet in their households in 2013, while the rate for the general population was 68% [24]. This situation will probably change with the next generation of computer-literate users reaching older age. However, as for now, the penetration of Internet use among the elderly remains much lower than in the average adult population. As a considerable number of people with disabilities belong to older groups of the general population, it is understandable that low Internet use among the population with disabilities may be partially attributable to their age. Consequently, the phenomenon of digital divide is particularly visible among the elderly with disability.

## Methods

### Overview

This paper was based on the analysis of data extracted from the results of a “Social Diagnosis” study performed in Poland in 2013. The study is a joint initiative of researchers gathered for the Council for Social Monitoring. Its main objective is the assessment of conditions and quality of life in Poland. The study began in the year 2000 and is carried out as a panel study and repeated every two years. 12,355 households were examined in 2013 and the number of individual respondents was 26,307 household members over 16 years of age. The data collected in consecutive waves of the study are available to interested users [7].

Households included in the survey are selected as the result of a two-stage stratified sampling. A detailed description of the sampling procedure and modifications introduced in consecutive waves of the study is provided in the relevant report [25].The survey was conducted by professional interviewers employed by the Central Statistical Office of Poland [26]. It was based on two questionnaires: the first focused on the household composition and living conditions; the latter on individual aspects of quality of life. The individual questionnaire was completed by all members of the household 16 years and over. Individual questionnaires were filled in the presence of the interviewer visiting each household included in the study. Each household member filled the questionnaire in confidentiality without the presence of other inhabitants of the household. The questionnaires used in the survey are available on the website of the “Social Diagnosis” study [7].

The “Social Diagnosis” study is performed according to ethical requirements and provisions independent from the analysis described in this paper. Research activities reported here relied on the secondary analysis of an anonymized dataset and did not require an additional consent from ethical committee.

### Data extraction

The analysis described in this paper was conducted on data originating from 3,556 individual questionnaires filled by household members who declared themselves as being disabled. Persons with disabilities can obtain specific support and relief in Poland on the condition that they have a confirmed status of disability. The disability status of the respondent could be established by three cases: if a respondent obtained a valid decision about their disability from the Disability Evaluation Board (DEB), had a decision about disability established for a child below 16 years of age or had a self-declared disability related to disease or a handicap.

The establishment of the status of disability and assessment of the grade of disability is conducted by a two-level Disability Evaluation Board (DEB). The Board can establish one of three grades of disability: mild, moderate or severe [27–28].

During the survey, household members who were Internet users, were asked to respond to questions on activities performed online. There were 965 people with disabilities in this subgroup of respondents. Their data were analyzed as to the frequency and factors influencing their undertaking specific activities online.

To summarize; the variables included in the analysis encompassed the use of the Internet and mobile telephony, items addressing socioeconomic status, a grade of disability and use of health care resources in the preceding year (hospital admission, services from healthcare provider, either public or private). In the subsample of respondents, who were Internet users, the variables reflecting specific activities performed on the Internet were used. Data set used in the analysis is available as S1 Dataset.

### Statistical analysis

Statistical analysis was performed using IBM SPSS v.21 (Armonk, NY, USA). The analysis reported in this paper was based on the data adjusted with calibrated weights provided by the authors of the “Social Diagnosis” study. The sample weighting system applied in the study is aimed at adjusting sample distortions resulting from the decrease of numbers of households and persons in subsequent panel studies related to refusals and loss of contact. Data collected within consecutive waves of the survey are weighted in order to maintain their national representation as well as for individual voivodships and class of the place of residence. Adjustments of initial weights were then applied according to refusals of joining the survey. The authors of the study applied integrated calibration to adjusted initial weights to provide weights simultaneously for households and their inhabitants. The calibration of adjusted initial weights was performed against external sources from the National Census of Population and Housing, and current demographic estimates. To summarize; the procedures of weighting was driven by the objective of assuring appropriate sample size and its representation on the national scale and in the cross-sections [25].

Descriptive analysis was carried out for the variables explored in the paper; frequencies were calculated for categorical variables. If not stated otherwise, the frequencies of responses to specific items were given as a percentage of all valid responses excluding missing responses. The assessment of predictors of being an Internet user and performing specific activities on the Internet was conducted with multivariate logistic regression modelling. A p level below 0.05 was treated as significant.

### Independent variables

The variables were selected according to their potential impact on the use of the Internet in the group of respondents with disability.

The severity of disability was established on the basis of grades assigned during assessment conducted by DEBs. The initial assumption was that, with growing severity of disability, Internet use will decrease. Most sociodemographic factors were selected on the basis of their availability in the set of variables included in the Social Diagnosis study and earlier reports on their influence on Internet use in other surveys [29–33]. The factors indicating the intensity of the utilization of health care resources were addressed, as preceding studies of the general population suggested that experiencing medical problems could influence Internet use or Internet use for searches of health-related information [34–39]. Previous reports from the Social Diagnosis waves tended to demonstrate that mobile telephony usage could be seen as a facilitator of Internet use in the general population [40]. Therefore, this factor was also addressed as a determinant of Internet use in the analysis.

In the result, on the basis of the items included in the individual version of the questionnaire used in the “Social Diagnosis” survey, twelve variables were established. The factors included grade of disability, sociodemographic characteristics (gender, age category, place of residence, education level [41], marital status, income status, net income category and socio-occupational status), the use of health care resources (hospital admission in preceding 12 months, the use of health care services in preceding 12 months) and the use of mobile phone.

### Dependent variables

The variable created on the basis of responses to the item asking about the use of the Internet, was used as a dependent variable in the logistic regression model. Furthermore, the activities performed online were assessed as to their frequency. Three variables based on performing online activities, were selected arbitrarily to represent various levels of IT skills and were also used as independent variables in the multivariate logistic regression models. They included accessing the website of public health institutions (low level skills), sending and receiving e-mails (medium level skills) and publishing personal content on the Internet (high level skills).

### Logistic regression modelling

Multivariate logistic regression was conducted by the forward method available in the SPSS v.21 package. For the independent variables included in the multivariate logistic regression models odds ratios (OR) and 95% confidence intervals (95% CI) were calculated.

As the percentages of missing values did not reach a level higher than 0.5% apart from the admission to hospital (S1 Table), the observations with at least one missing value in a variable included in logistic regression models were excluded from the analysis. In the result, the multivariate logistic regression model of Internet use was calculated, after adjusting for standardized weights from a dataset of 3,427 cases. The models of accessing websites of public institutions and publishing own content on the Internet were obtained from a datasets of 980 cases. Finally, the model of receiving and sending e-mails was based on a dataset of 981 cases.

Multivariate logistic regression modelling was preceded by multicollinearity diagnostic analysis with a calculation of variance inflation factor (VIF) values for independent variables. No concerns were raised, since all VIF values were below 2.0 (See S2 Table).

## Results

### Sample characteristics

In this paper, data from 3,556 respondents included in the 2013 wave of the “Social Diagnosis” study who confirmed being disabled and filled an individual questionnaire were included in the analysis. Weighted frequencies of study group characteristics are presented in Table 1. Females were 51.02%, and persons of 65 years of age and over 25.19% of all respondents. The degree of disability of 86.62% respondents was assessed and 22.02% were assigned to the severe disability group. As for occupational activities, 71.81% of respondents were retired or obtained disability pensions. The percentage of respondents who were professionally active was 14.07%. Finally, Internet users were 33.05% and those having mobile phone or a smartphone composed 73.27% of the study group.

**Table 1:** Characteristics of the study group (n = 3,556).

Variable	unweighted		weighted	
	n	%	n	%
**Degree of disability**				
mild degree	876	24.63	817	25.33
moderate degree	1364	38.36	1267	39.27
severe degree	914	25.70	783	22.02
not determined	402	11.30	359	11.13
**Gender**				
female	1907	53.63	1646	51.02
male	1649	46.37	1580	48.98
**Age**				
16–24 years	119	3.35	147	4.14
25–34 years	169	4.76	247	7.67
35–44 years	191	5.38	204	6.33
45–59 years	992	27.93	927	26.10
60–64 years	614	17.29	492	15.27
65+	1467	30.69	1204	25.19
**Place of residence**				
rural	1516	42.66	1141	35.39
urban <20,000	485	13.65	419	13.00
urban 20,000–100,000	787	22.14	779	24.16
urban 100,000–200,00	234	6.58	250	7.75
urban 200,000–500,000	306	8.61	314	9.74
urban >500,000	226	6.36	321	9.96
**Level of education**^**a**^				
primary education	1137	32.07	953	29.65
lower secondary education	1167	32.92	1071	33.32
upper secondary education	878	24.77	831	25.86
post-secondary non-tertiary education or higher level	363	10.24	359	11.17
**Occupational status**				
employee	345	9.74	405	12.60
self-employment or entrepreneur	28	0.88	26	0.81
farmer	26	0.73	21	0.65
retired or on disability pension	2740	77.38	2308	71.81
university or school student	67	1.89	91	2.83
unemployed	335	8.64	363	9.37
**Marital status**				
married	2028	57.27	1656	51.54
unmarried	531	15.00	617	19.20
widower/widow	742	20.95	699	21.76
divorced or separated	240	6.78	241	7.50
**Available source of income**				
not available	141	3.98	156	4.86
available	3399	96.02	3057	95.14
**Net income**				
<1000 PLN	1178	33.13	1027	31.83
from 1000 to <1500	968	27.22	851	26.37
from 1500 to <2000 PLN	564	15.86	509	15.77
> = 2000 PLN	411	11.56	419	12.98
not provided	435	12.23	421	13.05
**Hospital admission in last 12 months**				
no admissions	2681	76.53	2425	76.40
at least one admission	822	23.47	749	23.60
**Use of health care services in last 12 months**				
no use	158	4.45	178	5.52
at least one episode of use	3393	95.55	3046	94.48
**Mobile phone use**				
no	1049	29.66	858	26.73
yes	2488	70.34	2352	73.27
**Internet use**				
no	2526	71.32	2151	66.95
yes	1016	28.68	1062	33.05

^a^ education categories used in the survey were mapped to the levels distinguished in the ISCED classification from 2011 [41]

### Activities performed online

Detailed questions about activities performed online were answered by 965 respondents who filled individual questionnaire and were Internet users. Unweighted and weighted frequencies of responses according to specific types of activities are shown in supporting file (S3 Table).

Three of the most frequent activities indicated by Internet users were checking and sending e-mails (81.6%), use of an Internet communicator (67.6%), and voice over Internet (62.5%). The least frequent activities encompassed downloading of free software (15.1%), publishing the results of personal creativity, e.g. blogs on the Internet (27.9%) and participating in training and courses online (33.2%).

### Predictors of Internet use

Estimates of multivariate logistic regression of Internet use as an independent variable are shown in Table 2. Overall, the multivariate logistic regression model revealed adequate goodness-of-fit (Hosmer and Lemeshow test χ^2^ = 7.16, df = 8, p = .519, Nagelkerke R^2^ = 0.573). Internet use by people with disabilities depended on age category, place of residence, level of education, marital status, occupational status, net income, degree of disability, use of health services in preceding year, and having a mobile phone or smartphone. Gender, being admitted to a hospital during the preceding year and availability of income source did not influence the use of Internet.

The use of Internet was significantly lower in the older age categories. The odds ratios (OR) for comparison between the youngest and the oldest age categories (60–64 and 64 years old and over) were, respectively only 0.04 (95% CI 0.02–0.09, p < .001) and 0.01 (95% CI 0.004–0.02, p < .001). The inhabitants of cities were consistently more prone to use Internet than inhabitants of rural areas (OR from 2.00 to 3.04, p values < .001, for comparisons between rural and urban areas categorized according to growing population intervals). The level of education was also a very strong predictor of Internet use. Persons with university or at least post-high school education were 18.17 times more likely to use Internet than those with basic or lower education (p < .001). The difference in Internet use were statistically significant between married persons and unmarried (OR; 95% CI: 0.61; 0.44–0.91, p = .013) and between married and widowed persons (0.67; 0.47–0.96, p = .029). There were no differences between married persons and those divorced or living separated (0.75; 0.52–1.08, p = .116).

**Table 2:** Multivariate logistic regression model examining predictors influencing Internet use among people with disabilities (n = 3,427).

Variable	OR (95% CI)	p
**Degree of disability**		
mild disability		**.043**
moderate disability	0.84 (0.65–1.08)	.167
severe disability	0.68 (0.50–0.93)	**.017**
not determined	1.13 (0.76–1.69)	.547
**Gender**		
male/female	0.83 (0.66–1.04)	.101
**Age category**		
16–24 years		**< .001**
25–34	0.37 (0.18–0.77)	**.008**
35–44	0.41 (0.19–0.89)	**.025**
45–59	0.07 (0.03–0.15)	**< .001**
60–64	0.04 (0.02–0.09)	**< .001**
above 64 years	0.01 (0.00–0.02)	**< .001**
**Place of residence**		
rural		**< .001**
urban <20,000	2.11 (1.49–2.99)	**< .001**
urban 20,000–100,000	2.44 (1.82–3.26)	**< .001**
urban 100,000–200,00	3.04 (2.03–4.56)	**< .001**
urban 200,000–500,000	2.00 (1.34–2.94)	**< .001**
urban >500,000	2.68 (1.83–3.93)	**< .001**
**Level of education**		
primary education		**< .001**
lower secondary education	2.06 (1.48–2.87)	**< .001**
upper secondary education	6.17 (4.35–8.75)	**< .001**
post-secondary non-tertiary education or higher level	18.17 (11.70–28.21)	**< .001**
**Marital status**		
married		**.012**
unmarried	0.63 (0.44–0.91)	**.013**
widower/widow	0.67 (0.47–0.96)	**.029**
divorced or in separation	0.75 (0.52–1.08)	.116
**Occupational status**		
employee		**< .001**
self-employed or entrepreneur	0.28 (0.11–0.76)	**.013**
farmer	1.08 (0.35–3.33)	.898
retired or on disability pension	0.56 (0.40–0.79)	**.001**
university or school student	6.15 (1.54–24.65)	**.010**
unemployed	0.59 (0.37–0.93)	**.024**
**Availability of the source of income**		
not available/available	1.22 (0.68–2.19)	.512
**Net income**		
net income <1000 PLN		**< .001**
net income from 1000 to <1500 PLN	1.12 (0.83–1.53)	.457
net income from 1500 to <2000 PLN	1.86 (1.31–2.64)	**.001**
net income >2000 PLN	2.03 (1.38–2.97)	**< .001**
not provided	1.03 (0.68–1.56)	.881
**Admission to hospital in last 12 months**		
no admissions/at least one admission	1.04 (0.82–1.33)	.749
**Use of health care services**		
no use/at least one episode of use	1.87 (1.19–2.94)	**.007**
**Mobile phone**		
no/yes	4.02 (2.82–5.74)	**< .001**
**Constant**	0.374	.089

### Predictors of specific activities performed online

The multivariate logistic regression modelling was performed for accessing websites of public institutions, using e-mail service and publishing own content on the Internet (e.g. as a blog). These three types of online activities were treated as examples of increasing IT skills.

The variables affecting access of websites of public institutions included age category, place of residence, and net income (Table 3). There were also some significant differences between comparator and other categories in marital status, and grade of disability.

**Table 3:** Multivariate logistic regression model of accessing websites of public institutions, checking and sending e-mails and publishing own content on the Internet.

	accessing websites of public institutions^a^		checking and sending e-mails^b^		publishing own content on the Internet^c^	
Variable	OR (95% CI)	p	OR (95% CI)	p	OR (95% CI)	p
**Degree of disability**						
mild disability		.081		**.011**		**.009**
moderate disability	1.47 (1.05–2.04)	**.024**	1.71 (1.11–2.64)	**.015**	1.82 (1.22–2.70)	**.003**
severe disability	0.99 (0.64–1.54)	.956	2.54 (1.38–4.66)	**.003**	2.11 (1.27–3.52)	**.004**
not determined	1.32 (0.79–2.21)	.290	1.81 (0.86–3.79)	.117	1.85 (1.03–3.31)	**.039**
**Gender**						
male/female	1.10 (0.81–1.48)	.549	1.79 (1.18–2.72)	**.006**	0.96 (0.68–1.35)	.793
**Age category**						
16–24 years		**< .001**		< .001		**< .001**
25–34	0.82 (0.41–1.66)	.587	0.40 (0.08–2.05)	.272	0.69 (0.34–1.40)	.301
35–44	0.39 (0.18–0.85)	**.018**	0.19 (0.03–0.99)	**.050**	0.29 (0.13–0.64)	**.002**
45–59	0.25 (0.11–0.54)	**< .001**	0.07 (0.01–0.37)	**.002**	0.14 (0.06–031)	**< .001**
60–64	0.31 (0.13–0.75)	**.009**	0.05 (0.01–0.28)	**.001**	0.08 (0.03–0.21)	**< .001**
above 64 years	0.13 (0.05–0.33)	**< .001**	0.03 (0.01–1.19)	**< .001**	0.04 (0.01–0.12)	**< .001**
**Place of residence**						
rural		**< .001**		.057		**.029**
urban <20,000	2.15 (1.32–3.50)	**.002**	0.83 (0.45–1.54)	.559	1.72 (0.98–3.00)	.058
urban 20,000–100,000	2.56 (1.68–3.88)	**< .001**	1.31 (0.76–2.26)	.327	2.04 (1.27–3.27)	**.003**
urban 100,000–200,00	2.51 (1.49–4.22)	**.001**	2.20 (1.04–4.66)	**.039**	2.13 (1.18–3.84)	**.012**
urban 200,000–500,000	2.33 (1.37–3.96)	**.002**	1.13 (0.56–2.29)	.732	2.50 (1.37–4.56)	**.003**
urban >500,000	2.87 (1.70–4.86)	**< .001**	2.22 (1.03–4.78)	**.042**	1.92 (1.04–3.55)	**.036**
**Level of education**						
primary education		**< .001**		**< .001**		.417
lower secondary education	1.51 (0.83–2.74)	.176	0.88 (0.46–1.71)	.713	0.69 (0.37–1.30)	.252
upper secondary education	2.72 (1.50–4.95)	**.001**	1.98 (1.01–3.90)	**.048**	0.82 (0.43–1.56)	.548
post-secondary non-tertiary education or higher level	4.18 (2.17–8.05)	**< .001**	5.10 (2.15–12.09)	**< .001**	1.00 (0.49–2.00)	.989
**Marital status**						
married		.165		.092		.647
unmarried	0.88 (0.55–1.42)	.610	1.86 (0.90–3.85)	.094	0.78 (0.47–1.31)	.346
widower/widow	1.15 (0.62–2.08)	.638	0.91 (0.43–1.92)	.799	0.83 (0.37–1.87)	.647
divorced or in separation	1.78 (1.05–3.02)	.033	0.56 (0.30–1.07)	.079	1.20 (.66–2.17)	.546
**Occupational status**						
employee		.497		.255		.094
self-employed or entrepreneur	0.96 (0.29–3.16)	.945	0.29 (0.06–1.41)	.124	0.53 (0.12–2.26)	.388
farmer	6.13 (1.05–35.67)	**.044**	0.23 (0.03–2.01)	.184	0.11 (0.01–2.24)	.149
retired or on disability pension	1.10 (0.72–1.67)	.669	0.69 (0.39–1.22)	.202	0.86 (0.53–1.39)	.542
university or school student	0.87 (0.36–2.11)	.759	0.27 (0.05–1.60)	.148	0.48 (0.19–1.21)	.117
unemployed	1.09 (0.63–1.88)	.755	0.48 (0.22–1.06)	.069	0.44 (0.24–0.83)	**.011**
**Availability of the source of income**						
not available/available	1.24 (0.64–2.42)	.525	1.04 (0.39–2.77)	.935	1.29 (0.63–2.65)	.491
**Net income**						
net income <1000 PLN		**.001**		**.002**		**.001**
net income from 1000 to <1500 PLN	0.98 (0.62–1.53)	.912	0.97 (0.55–1.70)	.901	0.88 (0.53–1.47)	.633
net income from 1500 to <2000 PLN	0.83 (0.51–1.36)	.462	1.16 (0.62–2.16)	.652	0.43 (0.24–0.78)	**.005**
net income >2000 PLN	1.71 (1.04–2.80)	**.033**	3.34 (1.61–6.93)	**.001**	1.17 (0.67–2.02)	.587
not provided	0.54 (0.30–0.97)	**.038**	0.68 (0.32–1.43)	.309	0.39 (0.19–0.79)	**.009**
**Admission to hospital in last 12 months**						
no admissions/at least one admission	1.26 (0.91–1.76)	.170	1.40 (0.89–2.23)	.149	1.15 (0.79–1.68)	.474
**Use of health care services**						
no use/at least one episode of use	0.61 (0.32–1.18)	.142	0.40 (1.23–1.28)	.123	0.61 (0.31–1.22)	.162
**Mobile phone**						
no/yes	1.04 (0.53–2.07)	.905	2.78 (1.26–6.13)	**.011**	0.89 (0.39–2.02)	.772
**Constant**		.294		.051		.548

^a^ model characteristics for accessing websites of public institutions: n = 980, goodness-of-fit Hosmer and Lemeshow test, χ^2^ = 15.09, df = 8, p = .057, Nagelkerke R^2^ = .196

^b^ model characteristics for checking and sending e-mails: n = 981, goodness-of-fit Hosmer-Lemeshow test, χ^2^ = 10.86, df = 8, p = 0.21, Nagelkerke R^2^ = .316

^c^ model characteristics for publishing own content on the Internet, e.g. as a blog: n = 981, goodness-of-fit Hosmer and Lemeshow test, χ^2^ = 6.10, df = 8, *p* = .636, Nagelkerke R^2^ = .231.

The use of e-mail service was influenced by gender, age category, level of education, net income, degree of disability and the use of mobile phone (Table 3). Finally, publishing of own content on the Internet depended on age category, place of residence, net income and degree of disability (Table 3). There was also a statistically significant difference between respondents having an employed status and those who were jobless. However, the variable related to the occupational status as such did not have statistically significant impact on the outcome variable.

## Discussion

Each wave of the”Social Diagnosis” study yields a rich data set of information allowing for the analysis of determinants of IT use in various groups of users. In this paper, the factors determining the use of Internet and the undertaking of specific online activities by people with disabilities were assessed. Among 26,307 individual questionnaires collected during the wave of Social Diagnosis performed in 2013 there were 3,556 questionnaires filled by people having disability [25].The weighted rate of Internet use in this group was 33.1% which is much lower than in the general Polish population of 16 years of age and over. The summary report from the 2013 wave of Social Diagnosis study indicated that this value was 63.8% [40]. In turn, according to EUROSTAT, in 2013 the percentage of respondents who used Internet within the prior 12 months in Poland was 65% [2]. This difference is clearly related to the fact that the age structure of the population of people with disabilities is not representative for the whole society. The elderly (65 years and over) composed as much as 25.2% of the study group while the percentage of people below 45 years was only 13.5%. According to Eurostat, the proportion of the general population aged 65 and over in Poland was 14.4% in 2013 [42]Eurostat Populaiton by age group]. It should be remembered that Internet users did not surpass 18% in Poland or 39% in the European population of age 65 years and over in 2013 [3]. The digital divide observed among people with disability may also indicate that IT, and particularly Internet use, are oftentimes not perceived by society as a source of opportunity for the individuals with disabilities.

A considerable difference in Internet use between people with disabilities and general population was also frequently reported in the earlier stages of the World Wide Web’s development [4,20,43–45].The results reported by Kaye suggested that in 1998 Internet use among persons with work disability was only 9.9% and among those with no disability as much as 38.1%. An analysis of data from the National Household Travel Survey performed in the USA in 2001 revealed that among persons with travel-limiting medical conditions, the percentage of Internet users (during the preceding 6 months) was significantly lower than among persons without such medical conditions (32.6% vs. 70.3%). Persistence of this difference was confirmed in later studies. It is worth emphasizing that Internet access among consumers with disabilities reported by the Office of Communications (Ofcom) for the British population [46] was 65% which corresponds with the rate of Internet use of the general Polish population (persons 15 years old and over) reported in 2013 [3]. Therefore the gap in Internet use between people with disabilities and general population seen in various countries may be further aggravated due to differences in different nation’s progress in developing an information society, and Poland still lags behind leaders in this area.

The predictors of Internet use in the study group included the grade of disability, sociodemographic variables, economic and occupational status, use of public or private health care services (but not being admitted to hospital in preceding year) as well as the use of mobile phone or smartphone. Gender did not have significant impact on the use of the Internet by people with disabilities.

Internet usage was less probable in the case of people with severe disabilities when compared to people with mild disabilities. The impact of degree of disability on Internet use was described by Henshaw et al. from a group of disabled persons with hearing problems [47]. The results of the national survey reported by Gracia and Herrero indicated that Internet users had better self-rated health than non-users [48]. Finally, the Ofcom study revealed that people with disabilities with multiple impairments had the lowest level of access to the Internet [46].

People with disabilities belonging to the youngest category were consistently more likely to use Internet than persons belonging to older age categories. As for place of residence, living in an urban area significantly increased the odds of Internet use. The highest differences in Internet use observed were dependent on education level. The probability of the use of the Internet among people with disabilities who achieved at least a post-secondary level of education was nearly 18 times higher.

A significant influence on Internet use was also exerted by marital status. People with disabilities who were married were more prone to use the Internet than persons living alone (unmarried or widowed). People with disabilities, who were employed by others, were more inclined to use the Internet than the self-employed, unemployed, retired or those receiving a disability pension. Finally, availability of a source of income did not affect Internet use, but the level of income did. The probability of Internet use by persons classified in the two highest income categories was about two times higher than in the case of persons with the lowest income levels.

The impact of sociodemographic, economic and occupational factors on the use of the Internet by people with disabilities is obvious and corresponds to the trends seen in the general population. The results of an analysis reported by Sindhu on the data from Internet and Computer use Supplement of the Current Population Survey performed in 2000 in the USA revealed that educational status and living in a metropolitan area (apart from computer ownership and race not included in the “Social Diagnosis” study), exerted similar effects on Internet use as in our study [49].

Nearly the same predicting effects of variables included in this study, were also reported by Vicente and Lopez who studied the people with disabilities from ten European countries [50]. In their analysis, Internet use was related to younger age, higher education level, being employed, higher income level, as well as to masculine gender. The impact of gender on Internet use was not confirmed in our study. Lower use of Internet by the people with disabilities in the United States who were older, less educated and living in lower income households was described both in the study of Kaye in 2000 [43] and in the report published by the Pew Research Center in 2011 [4].

Significance of age and socioeconomic profiles was described in the population with disabilities in the UK [46]. Low income or unemployment was indicated among key barriers for obtaining Internet access by people with disability. The level of income achieved by the people with disabilities is all the more important as frequently they need to cover not only the costs of their terminals and connection subscriptions, but also access technologies required in case of specific types of disability [20]. The challenge of purchasing assistive technology devices or software, e.g. screen reader, apart from the standard costs of a computer or Internet access was also reported by [51]. Occupational status is an important predictor of Internet use not only because of its relation to economic status, but also because of the opportunity to have contact with computers and the Internet in the workplace [20].

Some authors underline that access to Internet became an issue of social justice and economic disadvantage and barriers to information access augment the effect of deprivation among people with disabilities, and especially those with intellectual disabilities [52]. It is also worth mentioning that in some countries there is a trend of narrowing digital divides. The OxIS report on Internet use in UK in 2013 indicated it diminished among individuals with disabilities but also for lower income groups. [53]

The utilization of health care resources had an unequivocal impact on Internet use. Generally, the use of public or private health care services led to increased probability of Internet use. Today, the Internet is a ubiquitous source of information about the health care system for most patients and citizens, thus, the necessity of obtaining health care service may motivate them to become Internet users. Paradoxically, admission to the hospital in the preceding year had no impact on being an Internet user, even if one might expect that more severe conditions requiring hospital care would lead to an increased need for health-related information searches on the Internet. On the other hand, hospitalization rates grow with more advanced age, so probably this effect is scaled down by the higher age of people with disabilities who had to be hospitalized. Unfortunately, in the 2013 wave of the Social Diagnosis study, there was no item asking about health-related Internet use.

Users of mobile telephony used the Internet more frequently than respondents who did not use mobile phones. Mobile telephony may be perceived as a driving force for Internet use among people with disabilities. Accumulated data from the Social Diagnosis study from years 2003–2013 demonstrates that the trend for mobile telephony use quickly outruns the use of computer and Internet. In 2013 the percentage of mobile telephony users in Poland was about 24% higher than the percentages of computer and Internet users [40]. Furthermore, persons who were simultaneously users of computer, Internet and mobile phone were 61% of the general population. One should also note that mobile phone users with a disability were 73.3% vs. 87.4% of the general Polish population. The difference is much lower than that seen in the case of the Internet use.

The most frequent online activity performed by persons with disabilities was checking and sending emails which was declared by nearly 82.0% of respondents who were Internet users in this group. The use of Internet communicators was second (about 68.0%). Other frequent activities included reading newspapers or books over the Internet, using social media (about 57.0%) and listening to music or radio over the Internet with frequencies in the range of 57.0–63.0%. A survey carried out in 2014 of the general Polish population revealed that the most frequent activity performed online by Internet users was searching (73%) [54]. Unfortunately this type of activity was not directly addressed in detail in the questionnaire used in the “Social Diagnosis” study. Using e-mail service was the second most frequent activity (71%), while watching video, reading news articles, using social media and listening to music were situated in the next positions with frequencies in the range of 51%-59%. The use of communicators was much lower in the general population (only 38%) than among Internet users with disabilities. This may support the notion that the Internet brings a new opportunity to this group of users and helps compensate for their mobility deficiencies or necessity to stay at home.

E-mail communication was the most frequent reason for Internet use in other studies focused on people with disabilities. Kaye’s report from 2000 revealed the same, with 67.1% of the Internet users with disabilities indicating e-mail service as such [43]. Sending e-mails was also the most common reason to use the Internet for older adults (65 years and over) in an analysis performed by Choi and DiNitto in 2013 taken from data of the National Health and Aging Trends Study based on the representative sample of US Medical beneficiaries [39]. The frequency of e-mail use among Internet users in this population did not differ considerably from that observed in the Polish sample of people with disabilities and reached about 86.0%.

The same set of independent variables for Internet use was assessed for predicting selected online activities. The influence of age category and net income was confirmed for all three analyzed online activities. The place of residence influenced accessing websites of public institutions and publishing own content on the Internet. In the case of sending and receiving e-mails, there were differences between inhabitants of rural areas and inhabitants of two categories of urban areas. The degree of disability influenced both using e-mails and publishing own content on the Internet. In the case of accessing websites of public institutions, the difference was statistically significant only for comparison of people with mild and moderate disability. Interestingly, more severe level disability favored publishing own content on the Internet. This effect may indicate that people with disabilities remaining mainly in their home environment are more determined to express themselves in the virtual world. The level of education exerted significant impact on the access of websites of public institutions and using e-mail services. Female users of mobile phones favored sending and receiving emails, but displayed no impact on the two other types of online activities. Contrary to Internet use, the use of health care services in preceding 12 months showed no influence on performing specific online activities.

This paper suffers from some limitations. The analysis performed in this paper did not encompass the specific types of disability. It seems that this could be an important factor influencing the use of Internet and performing specific activities online. One can expect that barriers to Internet use differ for people with disabilities with sensory deficits or with mobility impairment. Furthermore, the Social Diagnosis study did not address the use of the Internet for health-related purposes. So it is rather difficult to refer to many studies focusing on this aspect of online activity. Finally, the intensity of Internet use was not analyzed in detail and this feature could also be strongly dependent on factors related to type and degree of disability.

To summarize, the study confirmed that people with disabilities in Poland experience a significant digital divide. Furthermore, the predictors of Internet use among the population of people with disabilities are very similar to those of the general population and shared by people with disabilities in other countries. The phenomenon of digital divide observed among the Polish population of persons with disabilities is aggravated by the relatively slow uptake of IT in Polish society. Significant progress of Internet access was made only after Poland joined the European Union in 2004. Finally, people with disabilities who are active on the Internet undertake various types of activities. The determinants of specific online activities are more nuanced than factors predicting overall Internet use.

The Internet may be a source of opportunities for people with disabilities both in terms of accessing information and increasing social inclusion. However, it seems that current strategies aimed at improving Internet participation of this population are not fully effective on a national level. As a considerable part of population of people with disabilities is composed of older adults and elderly, programmes improving digital skills in these groups could also ameliorate digital divide among people with disabilities. Furthermore, a budget dedicated to people with disabilities and made available on national or regional levels could be directed to support purchases of computer equipment, reimbursement of Internet access costs and increase of IT skills. Finally, wide implementation of guidelines enabling easier access to Internet content to the persons with disabilities such as WAIG should be an element of public policy and made obligatory for websites run by public institutions.

## Supporting information

S1 Dataset Data set used for the analysis. (XLSX)

S1 Table Missing values. (DOCX)

S2 Table Collinearity testing results. (DOCX)

S3 Table Unweighted and weighted frequencies for activities performed online. (DOCX)
