# Supplementary material for: Digital divide among people with disabilities: Analysis of data from a nationwide study for determinants of Internet use and activities performed online
Source: PLoS One. 2017 Jun 29;12(6):e0179825. doi: 10.1371/journal.pone.0179825 (PMC5491040; doi:10.1371/journal.pone.0179825)
Supplement: S1 Table — (DOCX) [file pone.0179825.s002.docx]

Table 1. Frequencies of missing values in variables included in the multivariate logistic regression model of Internet use.

| Variable | n | % |
| --- | --- | --- |
|  |  |  |
| Internet use | 14 | 0.4 |
| grade of disability | 0 | 0 |
| Gender | 0 | 0 |
| age category | 4 | 0.1 |
| place of residence | 2 | 0.1 |
| level of education | 11 | 0.3 |
| marital status | 15 | 0.4 |
| available source of income | 16 | 0.4 |
| net income | 0 | 0 |
| socio-occupational status | 15 | 0.4 |
| use of health care services | 5 | 0.1 |
| hospital admission | 53 | 1.5 |
| mobile phone | 19 | 0.5 |
