# Supplementary material for: Digital divide among people with disabilities: Analysis of data from a nationwide study for determinants of Internet use and activities performed online
Source: PLoS One. 2017 Jun 29;12(6):e0179825. doi: 10.1371/journal.pone.0179825 (PMC5491040; doi:10.1371/journal.pone.0179825)
Supplement: S2 Table — (DOCX) [file pone.0179825.s003.docx]

Table 2. The results of collinearity testing for variables included in multivariate logistic regression model of Internet use.

| Variable | Tolerance | VIF |
| --- | --- | --- |
|  |  |  |
| grade of disability  gender  age category  place of residence  level of education  marital status  available source of income  net income  occupational status  use of health care services  admission to hospital  mobile phone | 0.951  0.918  0.766  0.860  0.769  0.915  0.666  0.768  0.802  0.947  0.968  0.800 | 1.051  1.090  1.305  1.163  1.301  1.093  1.501  1.303  1.247  1.056  1.033  1.250 |
