# Supplementary material for: Digital divide among people with disabilities: Analysis of data from a nationwide study for determinants of Internet use and activities performed online
Source: PLoS One. 2017 Jun 29;12(6):e0179825. doi: 10.1371/journal.pone.0179825 (PMC5491040; doi:10.1371/journal.pone.0179825)
Supplement: S3 Table — (DOCX) [file pone.0179825.s004.docx]

S3 Table. Unweighted and weighted frequencies for activities performed online

| Activities performed online | Unweighted | | Weighted | |
| --- | --- | --- | --- | --- |
|  | Yes | No | Yes | No |
|  | n (%) | n (%) | n (%) | n (%) |
| downloading free software | 134 (13.9) | 831 (86.1) | 154 (15.1) | 865 (84.9) |
| publishing own content (e.g. blog) | 236 (24.5) | 729 (75.5) | 284 (27.9) | 735 (72.1) |
| courses or training online | 278 (28.8) | 687 (71.2) | 338 (33.2) | 681 (66.8) |
| creating own website | 294 (30.5) | 671 (69.5) | 356 (34.9) | 663 (65.1) |
| occupational activities | 316 (32.7) | 649 (67.3) | 385 (37.8) | 634 (62.2) |
| ticket booking | 329 (34.1) | 636 (65.9) | 382 (37.5) | 637 (62.5) |
| participation in Internet auction | 333 (34.5) | 632 (65.5) | 389 (38.2) | 630 (61.8) |
| job search | 374 (38.8) | 591 (61.2) | 450 (44.2) | 569 (55.8) |
| discussion fora | 385 (39.9) | 580 (60.1) | 454 (44.5) | 565 (55.5) |
| downloading free music or videos | 401 (41.6) | 564 (58.4) | 472 (46.3) | 547 (53.7) |
| participation in chat | 412 (42.7) | 553 (57.3) | 492 (48.3) | 527 (51.7) |
| downloading or filling in administrative forms | 412 (42.7) | 553 (57.3) | 464 (45.5) | 555 (54.5) |
| network gaming | 417 (43.2) | 548 (56.8) | 487 (47.8) | 532 (52.2) |
| watching TV, video over Internet | 455 (47.2) | 510 (52.8) | 512 (50.2) | 507 (49.8) |
| accessing websites of public institutions | 456 (47.3) | 509 (52.7) | 498 (48.9) | 512 (51.1) |
| collecting materials needed for learning or work | 470 (48.7) | 495 (51.3) | 560 (54.9) | 459 (45.1) |
| Internet banking | 513 (53.2) | 452 (46.8) | 575 (56.4) | 444 (43.6) |
| purchases online in Poland and abroad | 518 (53.7) | 447 (46.3) | 578 (56.8) | 441 (43.2) |
| listening to music or radio over the Internet | 538 (55.8) | 427 (44.2) | 618 (60.7) | 401 (39.3) |
| using Facebook and/or other social media | 553 (57.3) | 412 (42.7) | 616 (60.4) | 403 (39.6) |
| reading newspapers or books over Internet | 558 (57.8) | 407 (42.2) | 581 (57.0) | 438 (43.0) |
| voice over IP, Skype | 592 (61.3) | 373 (38.7) | 637 (62.5) | 382 (37.5) |
| using Internet communicators | 628 (65.1) | 337 (34.9) | 689 (67.6) | 330 (32.4) |
| checking and sending emails | 771 (79.9) | 194 (20.1) | 832 (81.6) | 187 (18.4) |
